# Borderline tumors of the ovary: A clinicopathological study

**DOI:** 10.12669/pjms.332.11847

**Published:** 2017

**Authors:** Samia Yasmeen, Abdul Hannan, Fareeha Sheikh, Amir Ali Syed, Neelam Siddiqui

**Affiliations:** 1 Dr. Samia Yasmeen, MBBS, FCPS (Medicine), Fellow Medical Oncology, Dept. of Medical Oncology, Shaukat Khanum Memorial Cancer Hospital & Research Centre, 7-A block, R-3 Johar Town, Lahore, Pakistan; 2 Dr. Abdul Hannan, MBBS, MD, FCPS (Medicine), FCPS (Medical Oncology), Resident Internal Medicine, East Tennessee State University Department of Internal Medicine, Johnson City, Tennessee Johnson City, USA; 3 Dr. Fareeha Sheikh, MBBS, FCPS(Medicine), Fellow Medical Oncology, Dept. of Medical Oncology, Shaukat Khanum Memorial Cancer Hospital & Research Centre, 7-A block, R-3 Johar Town, Lahore, Pakistan; 4 Dr. Amir Ali Syed, MBBS, FRCS, Consultant Surgical Oncologist, Dept. of Surgical Oncology, Shaukat Khanum Memorial Cancer Hospital & Research Centre, 7-A block, R-3 Johar Town, Lahore, Pakistan; 5 Dr. Neelam Siddiqui, MBBS, FRCP, CCST (Medical Oncology), Consultant medical Oncologist, Dept. of Medical Oncology, Shaukat Khanum Memorial Cancer Hospital & Research Centre, 7-A block, R-3 Johar Town, Lahore, Pakistan

**Keywords:** Borderline ovarian tumor, FIGO, Mucinous ovarian tumor, Serous ovarian tumor

## Abstract

**Objective:**

To report experience with borderline ovarian tumors (BOTs) in a developing country like Pakistan with limited resources and weak database of health system.

**Methods:**

Patients with BOTs managed at Shaukat Khanum Cancer hospital, Lahore, Pakistan from 2004 to 2014 were included and reviewed retrospectively. Data was recorded on histopathological types, age, CA-125, stage of disease, treatment modalities and outcomes.

**Results:**

Eighty-six patients with BOT were included with a median age of 35 years. Forty-two (49%) patients had serous BOTs and 43 (50%) had mucinous BOTs, while one (1%) had mixed type. Using FIGO staging, 80 patients had stage I; two patients had IIA, IIB and stage III each. Median follow-up time was 31.5 months. All patients had primary surgery. Seventy (81%) patients underwent complete surgical resection of tumor. Forty-three (50%) patients had fertility preserving surgery. Seventy-three (85%) patients remained in remission. Recurrent disease was observed in 13 (15%) patients. Median time to recurrence was 22 months. On further analysis, age above forty years, late stage at diagnosis and incomplete surgery were significantly associated with invasive recurrence.

**Conclusion:**

Despite a low malignant potential, relapses may occur in patients above forty years of age, incomplete surgery and staging information and advanced stage at presentation. Fertility sparing surgery should be considered in young patients. Complete excision of tumor and prolonged follow-up are advised because recurrence and transformation to invasive carcinoma may occur.

## INTRODUCTION

Borderline ovarian tumours, (BOTs) are recognised as a unique entity of ovarian neoplasms that characteristically lack stromal invasion, which is a hallmark of invasive cancers.^1^-^3^ BOTs have been referred by different names like borderline or atypical proliferative as well as tumours of low-malignant potential.

BOTs comprise of 10-20% of all ovarian neoplasms.^4^ The incidence of BOTs is 1.8–4.8 cases per 100,000 females per year.^5^ They typically have an excellent prognosis with an overall 10 year survival rate of 83-91%.^6^,^7^ They are predominantly diagnosed in pre-menopausal females aged 34–40 years and are rare in women who are 65 years of age or older.^5^ Histopathologically, this group of neoplasms exhibit behaviour that is intermediate between benign cyst-adenomas and invasive carcinomas. Two subtypes of BOTs, serous and mucinous borderline tumors are more common and have different characteristics as well as different clinical behaviour.^8^,^9^ Rarely other types like endometroid, clear-cell or transitional cell (Brenner) borderline tumors are also present.^10^

Surgery remains the ideal treatment for patients with BOTs. Two standard methods used are conservative or radical surgery depending upon the age, child bearing and menopausal status as well as the desire of fertility preservation among individual patients and most importantly the histological characteristic of the tumor.^11^-^15^

Comprehensive staging surgery includes total abdominal hysterectomy, omentectomy, peritoneal washings, bilateral salpingo-oophorectomy and various biopsies, including pelvic and para-aortic lymph node sampling.^16^,^17^ Since BOTs often occur in patients during their childbearing years, numerous studies have proposed fertility sparing surgery for those patients who desire to retain their reproductive function. Neither chemotherapy nor radiotherapy is currently accepted as part of the standard care. Chemotherapy is considered only in cases of peritoneal invasive implants.^16^

Due to rarity of disease and lack of centralised cancer registries very limited published data is available from third world countries. In this study, we present the characteristics, behaviour and treatment outcomes of BOTs from a single tertiary care cancer hospital in Pakistan over a period of 10 years from 2004 to 2014.

## METHODS

This is a retrospective study. All patients presenting with a diagnosis of BOTs at SKMCH and RC, Lahore from January 2004 to December 2014 were studied. Data was retrieved from the hospital computer registry and all computerised and paper based records were reviewed. Final cut off for chart review was December 2014. Only those patients were included in this study that were treated and followed up at SKMCH and RC, Lahore. Those patients who were lost to follow up after initial presentation or were not treated at SKMCH and RC were excluded. All personal identifiers were removed and data was retrieved on a structured questionnaire containing age, type of tumour, CA-125, stage, treatment, outcome and follow up details.

Before embarking on treatment, the histopathology was reviewed and classified according to WHO classification (mucinous, serous, mixed or others). Staging was done using International Federation of Gynaecology and Obstetrics (FIGO staging system). Treatment details were collected and grouped according to type of surgery, whether fertility sparing or non-fertility sparing, as well as complete or incomplete surgical staging.

The status of disease when last seen at our hospital was classified into two categories i.e. remission (no disease) and residual/recurrent disease. Recurrent disease was further categorized as recurrence of tumor with BOTs features or invasive recurrence.

Data was analysed using Statistical Package for Social Sciences software (SPSS) version 20. Descriptive analysis was done using mean, median and standard deviation for continuous variables like age, median survival in months and frequencies/percentages for categorical variables like FIGO staging and disease status.

## RESULTS

Eighty-six (86) patients with BOTs were identified through the hospital cancer registry. Descriptive Statistics of sampled population with BOTs including median age, CA-125 value, histological characteristics of tumors and complete staging information based on FIGO staging system are shown in Table-I.

**Table-I:** Descriptive Statistics of sampled population with borderline ovarian tumours (n=86).

*Characteristics*		*Count (Percentage)*
Age (yrs.)	Median	35 (26-45)
CA 125 (0-35U/ml)	Median	21 (13-62)
Histological type of tumour	Mucinous	43 (50%)
	Serous	42 (49%)
	Mixed	1 (1%)
FIGO Staging	1A	44 (51%)
	1B	21 (25%)
	1C	15 (18%)
	II-IV	6 (6%)

Seventy (81%) of the patients underwent complete surgical resection of tumor as per ovarian cancer surgery protocol. The remaining sixteen (19%) patients, who had incomplete surgical resection, included those who had their first surgical procedure at an outside facility and were not willing for second surgery. These patients were kept on clinical follow-up. Forty-three (50%) patients underwent fertility preserving surgery.

During the median follow-up time of 31.5 months (3-114 months), remission was achieved in 73 (85%) patients and recurrent disease was observed in 13 (15%) patients, of which five patients had invasive recurrence and eight had borderline recurrence.

Five patients (6%) had invasive recurrence (one patient had stage 1A, two patients with stage 2B and two patients with stage 3A). None of them had received complete surgical staging, while two patients had fertility sparing surgery.

All of these patients with invasive recurrence were later treated with chemotherapy. Two of them died during treatment, two patients were alive with disease and only one patient had complete response to chemotherapy.

Among eight patients (9%) with borderline recurrence (five patients with stage 1A, one patients with stage 1B and two patients with stage 1C), only two patients underwent complete surgical staging and rest of the six patients had incomplete surgical staging. Fertility sparing surgery was done in five of these patients. Six patients underwent second surgical procedure for recurrence and became disease free after that. Rest of the two patients were offered repeat surgical procedure but they were lost to follow-up. Disease status of the sampled population having details on remission, recurrence and type of recurrence based on the tumor histology is described in Table-II.

**Table-II:** Type of tumor and disease status (n=86).

			*Disease Status*		*Total (n)*

		*Invasive Recurrence (n)*	*Borderline Recurrence (n)*	*Remission (n)*
Type of tumor	Mucinous borderline ovarian tumor	3 (6.82%)	3 (6.82%)	38 (86.36%)	44
	Serous borderline ovarian tumor	2 (4.88%)	5 (12.91%)	34 (82.93%)	41
	Mixed borderline ovarian tumor	0	0	1 (100%)	1
		5 (5.81%)	8 (9.30%)	73 (84.88%)	86

Six patients (7%) with disease in remission conceived spontaneously after fertility sparing surgery for borderline ovarian tumors with uneventful pregnancies and child births. On multivariate analysis, factors significantly associated with invasive recurrence are; age above forty years, incomplete surgery and staging information and advanced stage at presentation. (Table-III)

**Table-III:** Multivariate logistic regression analyses to explore the association of factors associated with recurrence.

	*Odds Ratio*	*95% Confidence interval*	*P*	*Adjusted odds Ratio*	*95% Confidence interval*	*p*
Age 40 years or older	3.3	0.96-11.1	0.06	11.2	2.55-48.9	<0.01
FIGO stage II or III (Compared to I)	15.8	2.49-99.8	<0.01	10.5	2.31-47.5	<0.01
Received fertility sparing surgery	1.2	0.36-3.94	0.73	3.36	0.65-17.3	0.15
Received completion surgery	0.03	0.005-0.13	<0.01	0.03	0.005-0.22	<0.01

## DISCUSSION

Borderline ovarian tumors (BOTs), though rare in clinical practice are extremely important from treatment and prognostic point of view, because if detected and treated early, these are curative malignancies. As they behave in between benign and malignant tumors, if treated at initial stages are almost always curative, with survival estimates of almost 100%.^5^ Only a little data has been published on BOTs from South East Asia and with small number of patient population. In our paper, we are reporting 10 years’ experience with BOTs from SKMCH and RC, Lahore, Pakistan. Overall 86 patients with BOTs were identified with a median age of 35 years. The median age when compared to international data was almost similar but surprisingly age range was quite wide in other studies as Loizzi et al.,^5^ reported age range of 13-79 years while Romeo et al,^16^ reported age range of 30-63 years. In our study, the range was only 26–45 years, demographic or study population differences may contribute but no specific factors were identified regarding this discrepancy.

Most of the BOTs are serous or mucinous type, while others like mixed, endometroid or clear cell types are rare. We identified only one patient with mixed type BOTs in our data. Regarding tumor histology, our data has almost equal number of both serous (49%) and mucinous (50%) histologies. Interestingly, histologic subtypes are different according to geographic location. Serous BOTs are more common in western countries while mucinous histologies are more commonly described in Asian population.^18^

Most of the patients in our study cohort presented at an early stage (80% at stage 1). Results are similar with regard to the surgical treatment and post-surgical outcomes as 86% (73 patients) were in remission at follow up. It is important to optimise and carefully examine the specimens for invasive implants anywhere in peritoneal cavity, as they may compromise the prognosis, in the form of recurrence. As these tumors occur in relatively younger patients as compared to their invasive counterparts, fertility is also a major issue in management of these malignancies. Unfortunately, in only 50% of our patients, fertility could be preserved. As our centre caters for patients from many other non-oncological centres and general hospitals, many of these surgeries were performed outside, compromising the fertility and were again operated for re-do surgeries. Literature comparing the fertility sparing and radical surgeries in BOTs suggest that the fertility sparing surgeries have consistently shown to have higher rates of recurrences.^6^ On multivariate logistic regression analysis, we identified that those patients who are older (> 40 years) and underwent incomplete surgeries have higher chances of recurrence as compared to younger patients and those who underwent completion surgeries (Table-III). However, we suggest that in a low to middle income country like ours with limited health care resources, the decision for fertility preservation should be reviewed carefully with full involvement of patients, considering their understanding of disease, risk of recurrence, menopausal status and desire of fertility.

Recurrences are rare if initial surgeries are complete with careful examination of the peritoneal cavity. We identified a total of 13 cases of recurrences, out of which 5 were invasive while 8 were borderline.^19^-^21^ Recurrence rates of 0-30% has been documented in literature, however recurrences are common, up to 19% in patients who underwent fertility sparing surgeries. It is important to emphasize that no survival differences have been noted in literature between patients who undergo complete or fertility sparing surgeries.^22^,^23^ Patients who have a recurrence may undergo a fertility sparing surgery again. In their study, Loizzi et al. reported that total recurrence rate of BOTs were 8.5% and cancerous transformation rates 5.4%. Among 11 relapse cases, 4 were borderline and 7 were malignant recurrence.^5^

Use of adjuvant chemotherapy for BOTs and especially in case of invasive recurrence remains controversial. No study has proven that adjuvant chemotherapy is beneficial but usually it is the treating oncologist choice to treat with chemotherapy in case of invasive recurrences especially with presence of un-resectable disease.^24^

### Limitations of the study

Our study is retrospective with small number of patients from a single centre, which is the major limitation of the study. Our results are compromised in terms of fertility or non-fertility sparring surgeries but survival results are at par with western data, which also emphasize need for treatment at tertiary care cancer hospitals and more education among gynaecologists, who are mostly the first physicians the patient encounters.

## CONCLUSION

Patients with BOTs present at a younger age compared to invasive epithelial cancer of the ovary. Despite a low malignant potential, relapses were noted possibly due to incomplete surgery and staging information, patients above forty years of age and advanced stage at presentation. Complete excision of tumor and prolonged follow-up are advised because recurrence and transformation to invasive carcinoma may occur.

### Authors’ Contribution

**SY & NS:** Conceived, designed and writing / editing of manuscript.

**AH & FS:** Data collection and manuscript writing.

**NS & AAS:** Review, editing and final approval of manuscript.

**SY:** Statistical Analysis.
